# Supplementary material for: Macrophage-derived GPNMB trapped by fibrotic extracellular matrix promotes pulmonary fibrosis
Source: Commun Biol. 2023 Feb 2;6:136. doi: 10.1038/s42003-022-04333-5 (PMC9893197; doi:10.1038/s42003-022-04333-5)
Supplement: Supplementary file 2 — Description of Additional Supplementary Data [file 42003_2022_4333_MOESM2_ESM.docx]

**Description of Additional Supplementary Files**

**File name:** Supplementary Data 1

**Description:** Additional figures and tables
