# Supplementary material for: Macrophage-derived GPNMB trapped by fibrotic extracellular matrix promotes pulmonary fibrosis
Source: Commun Biol. 2023 Feb 2;6:136. doi: 10.1038/s42003-022-04333-5 (PMC9893197; doi:10.1038/s42003-022-04333-5)
Supplement: Supplementary file 3 — Supplementary Data 1 [file 42003_2022_4333_MOESM3_ESM.pdf]

Macrophage-derived GPNMB trapped by fibrotic extracellular matrix promotes pulmonary fibrosis

Online supplement

Supplementary Figure S1	2-3
Supplementary Figure S2	4-5
Supplementary Figure S3	6-7
Supplementary Figure S4	8
Supplementary Figure S5	9-10
Supplementary Figure S6	11-12
Supplementary Figure S7	13
Supplementary Figure S8	14
Supplementary Figure S9	15
Supplementary Figure S10	16
Supplementary Figure S11	17
Supplementary Figure S12	18
Supplementary Figure S13	19
Supplementary Figure S14	20
Supplementary Figure S15	21
Supplementary Table S1	22-23
Supplementary Table S2	24

Supplementary Figure 1

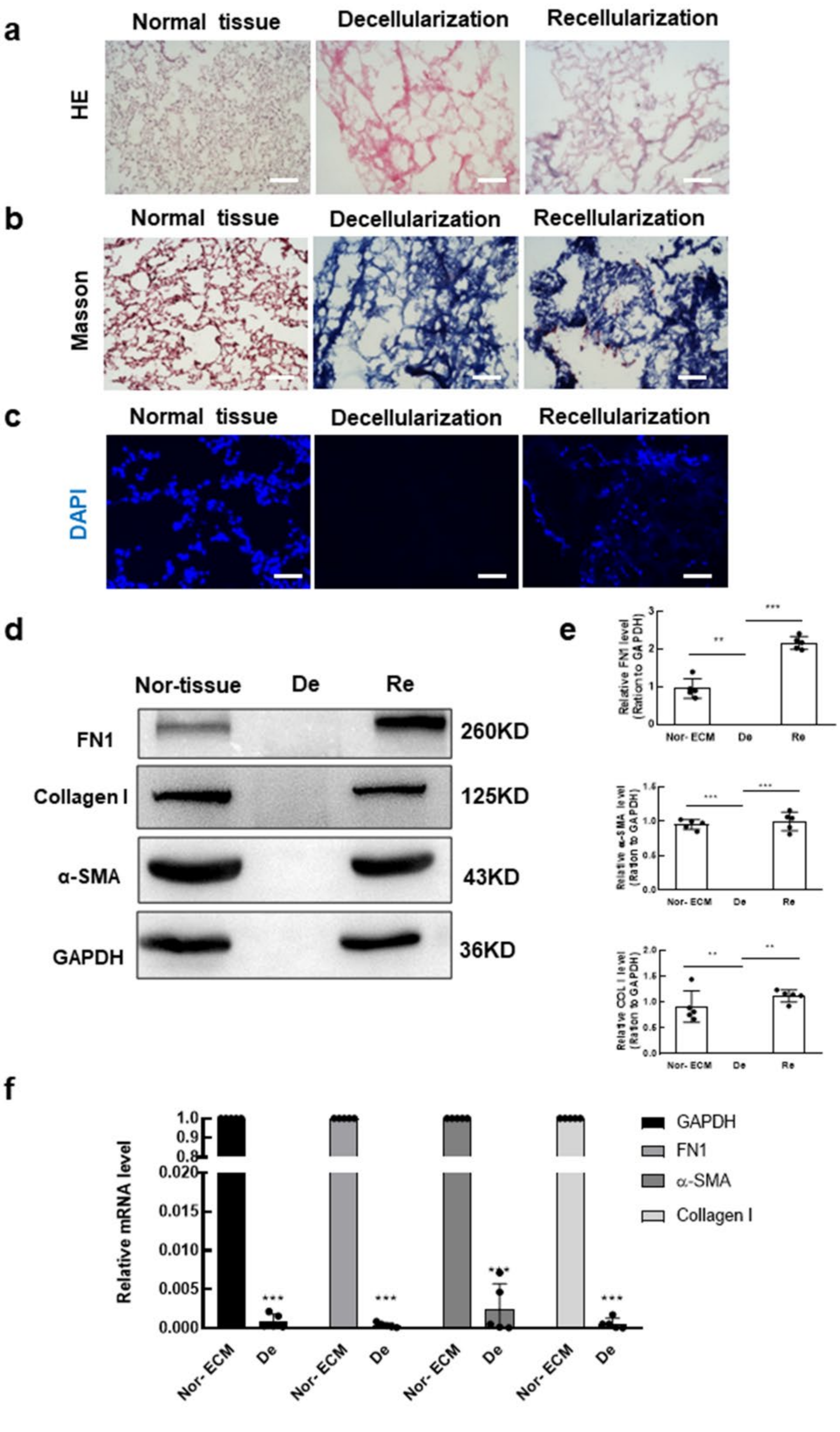

Supplementary Figure 1. Extraction and identification of mouse lung ECM. a-b H&E and Masson's trichrome staining of normal lung tissue, decellularized normal lung tissue (ECM) and ECM following cell repopulation. n=5, scale bar=200 μm. **c** DAPI staining of normal lung tissue, decellularized normal lung tissue (ECM) and ECM following cell repopulation. n=5, scale bar=200 μm. **d-e** Western blot analysis of cellular protein expression in normal lung tissue, decellularized normal lung tissue (ECM) and ECM following cell repopulation. n=5, ***p<0.01, **p<0.001. **f** qRT–PCR analysis of the expression of various housekeeping genes in nondecellularized lung and decellularized lung matrix, n=5, ***p<0.01, **p<0.001.

Supplementary Figure 2

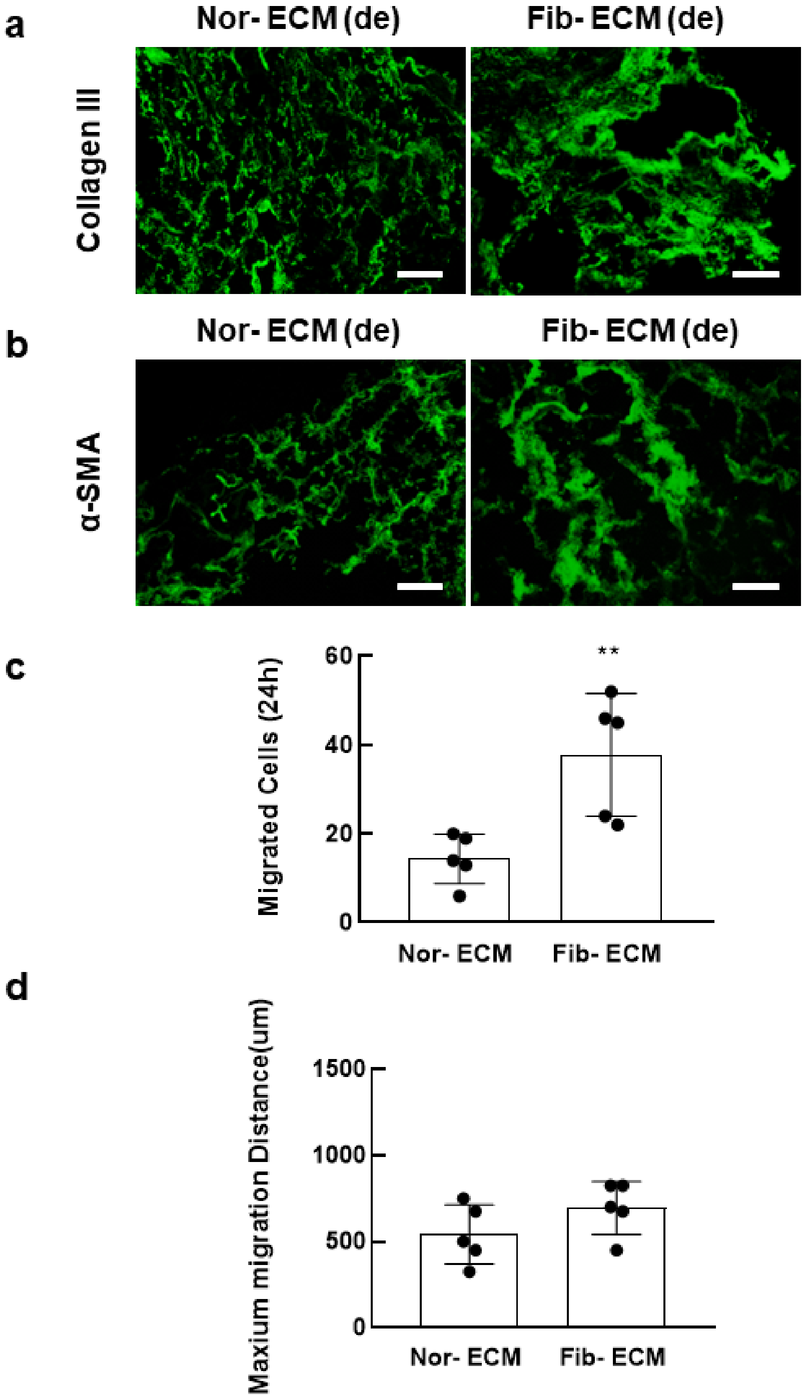

Supplementary Figure 2. Changes of lung ECM in mice after silica infusion. a-b Analysis of changes in ECM components after silica instillation by immunofluorescence staining. n=5, scale bar=200 μm. **c** The number of cells in each field that migrated from the nested matrix; n=5, $**p<0.01$. **d** The maximum migration distance per field was averaged; n=5, $**p<0.01$.

Supplementary Figure 3

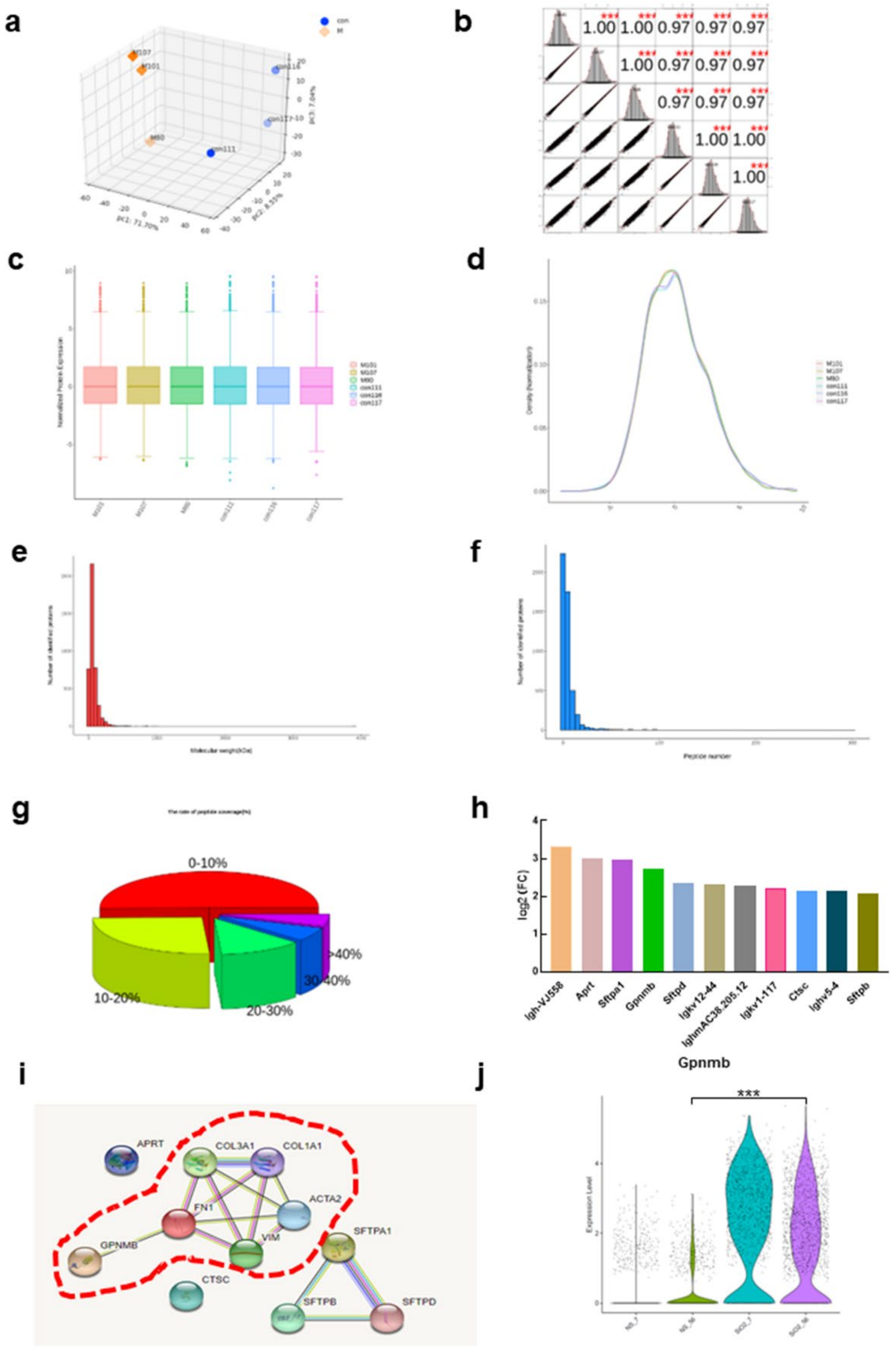

Supplementary Figure 3. Increased GPNMB in the fibrotic ECM involved in PF. a Principal component analysis. **b** Sample correlation analysis. **c** Visualization of the data before and after standardization. **d** Density plot. **e-g** Qualitative statistics. **h** The top genes corresponding to the upregulated proteins in ECM. **i** Protein interaction network analysis between GPNMB and fibrosis-related proteins. **j** GPNMB levels in different groups.

Supplementary Figure 4

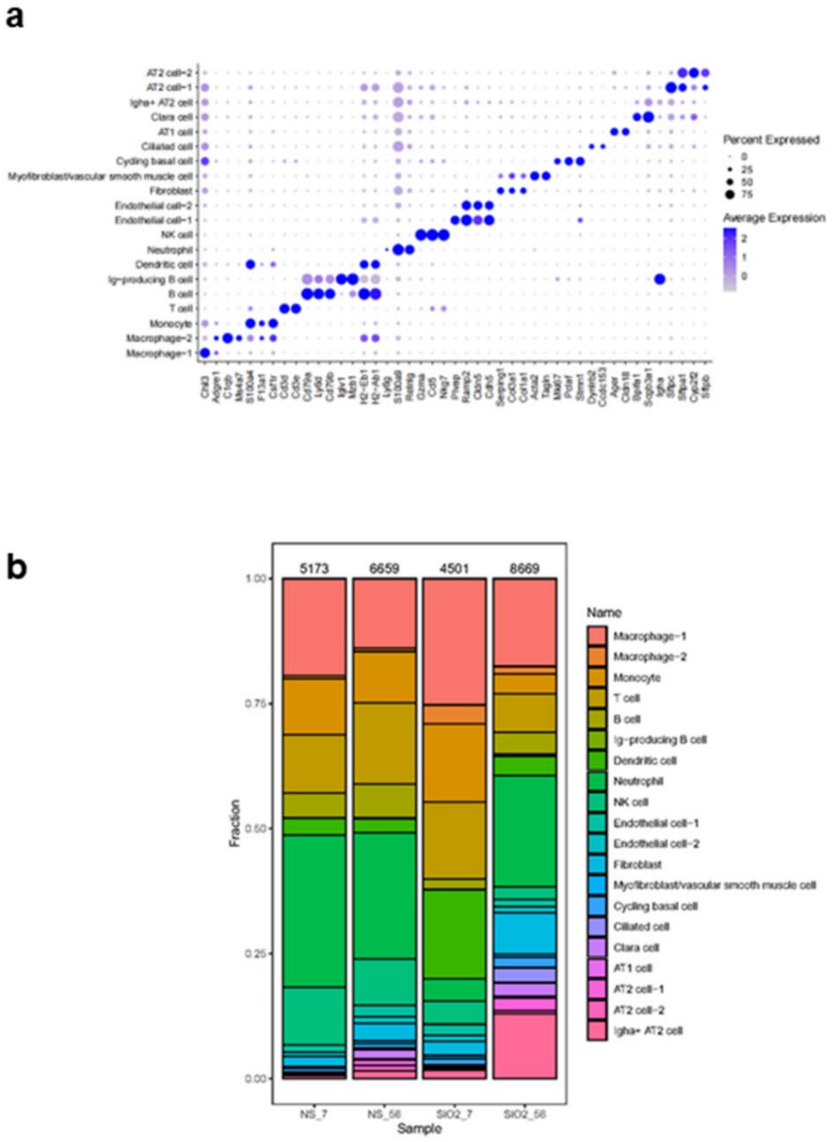

Supplementary Figure 4. The expression of cell markers and proportions of each cell type according to the scRNA-seq data. a The expression of cell markers in the 20 cell types. **b** Proportions of each cell type obtained from samples. Each cell type is labeled with a specific color.

Supplementary Figure 5

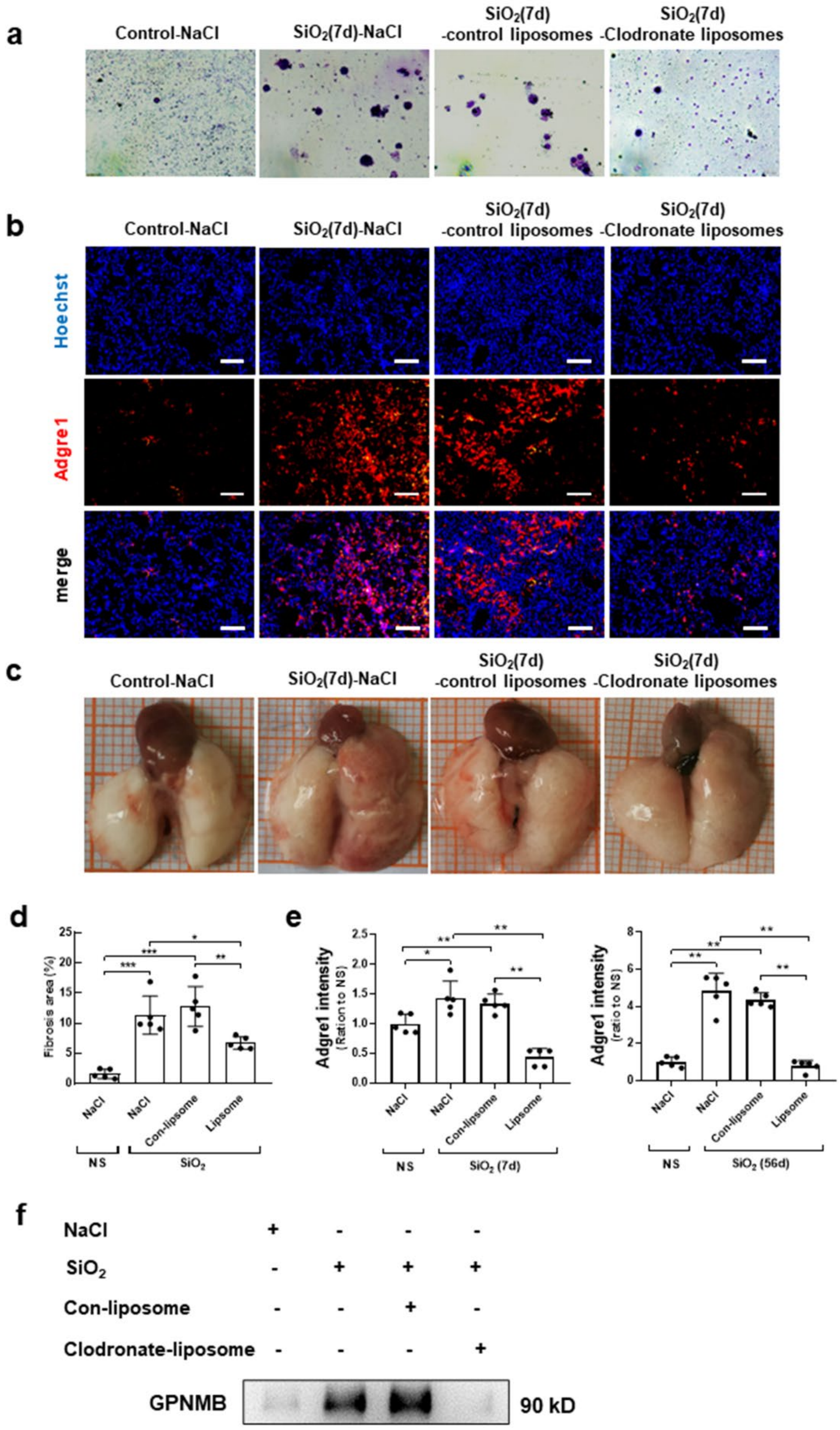

Supplementary Figure 5. ECM changes in macrophage-deletion mice. a Wright staining showed that the number of macrophages was decreased in the alveolar lavage fluid of silicosis mice after treatment with clodronate-containing liposomes. **b** Immunofluorescence staining showed that the number of macrophages was decreased in the lungs of silicosis mice after treatment with clodronate-containing liposomes. n=5, scale bar=200 μm. **c** Macrophage deletion decreased the severity of lung injury induced by silica. **d** Masson's trichrome staining showed that the fibrotic area of the lungs was decreased after macrophage deletion. n=5, *p<0.05, **p<0.01, ***p<0.001. **e** Quantification for Immunofluorescence staining of mouse lung tissue sections from the four groups, n=5, *p<0.05, **p<0.01. **f** Representative Western blot of GPNMB in ECM from the four groups, n≥3.

Supplementary Figure 6

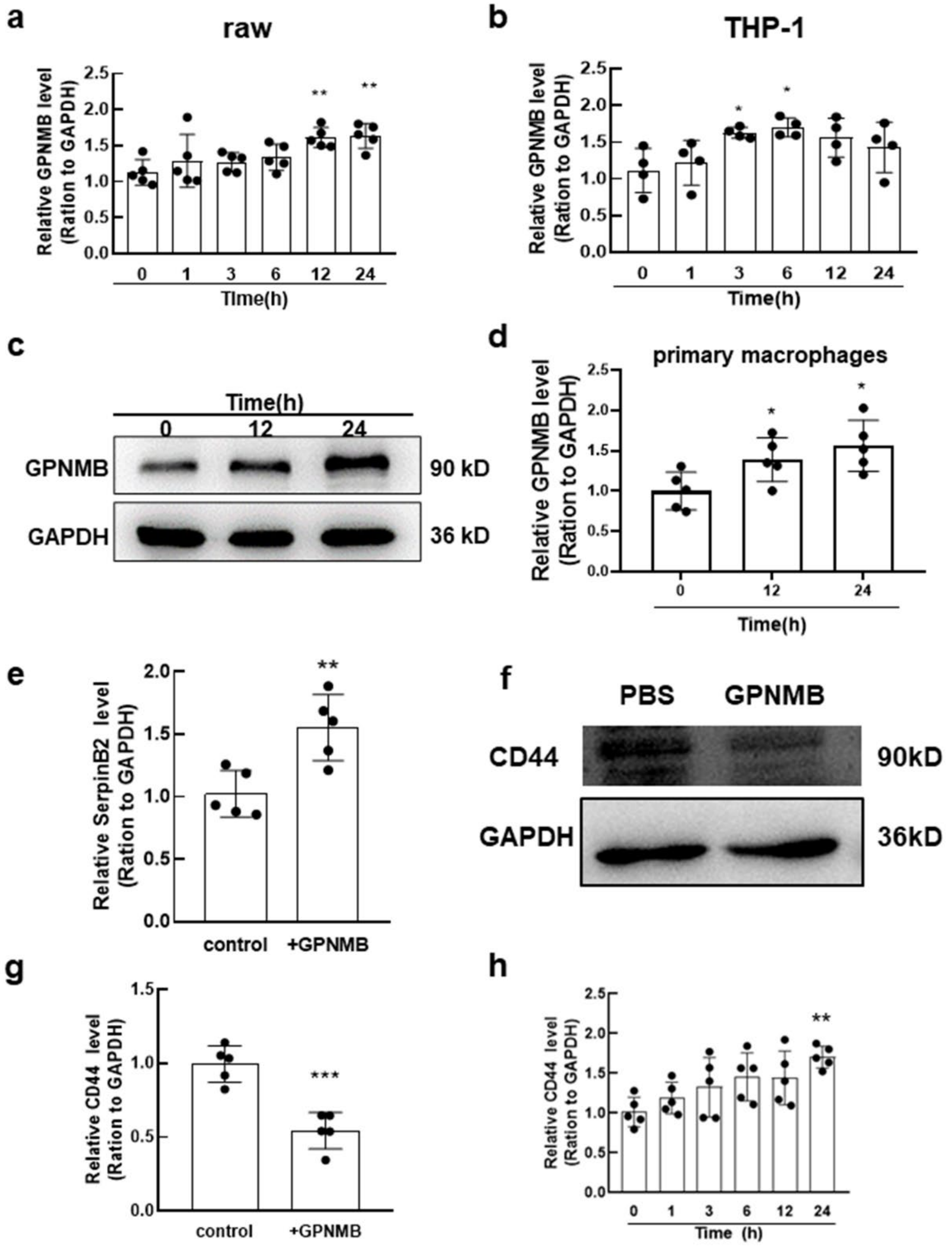

Supplementary Figure 6. GPNMB/CD44/Serpinb2 involved in fibroblast activation. **a** The GPNMB protein level in RAW264.7 cells was increased after silica stimulation; n=5. **b** The GPNMB protein level in THP-1 cells was increased after silica treatment; n=5. **c-d** The GPNMB protein level in primary macrophages was increased after silica stimulation; n=5, **p<0.01. **e** Western blot analysis showed that Serpinb2 levels were increased in fibroblasts treated with GPNMB; n=5, **p<0.01. **f-g** Western blotting showed that the CD44 protein level was decreased after GPNMB treatment; n=5, **p<0.001. **h** Western blot analysis showed that CD44 levels were increased in fibroblasts treated with TGFβ1; n=5, **p<0.01.

Supplementary Figure 7

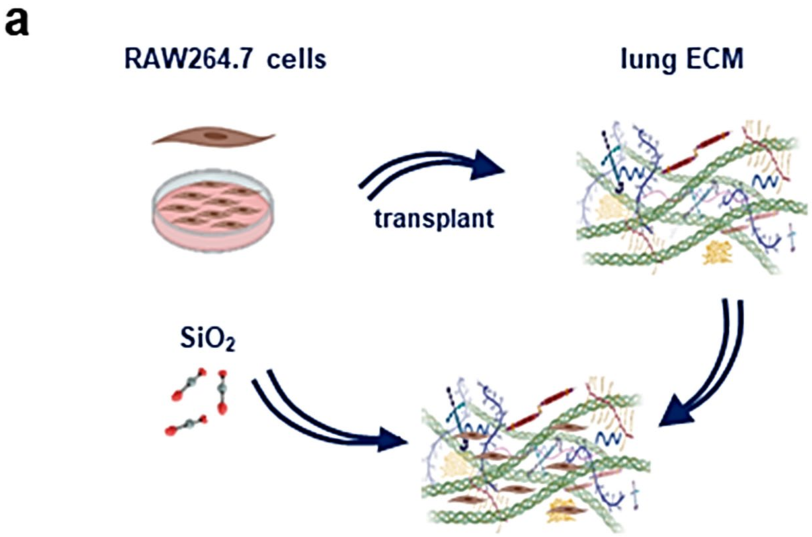

Supplementary Figure 7. Experimental process for coculture of macrophages and ECM

Supplementary Figure 8

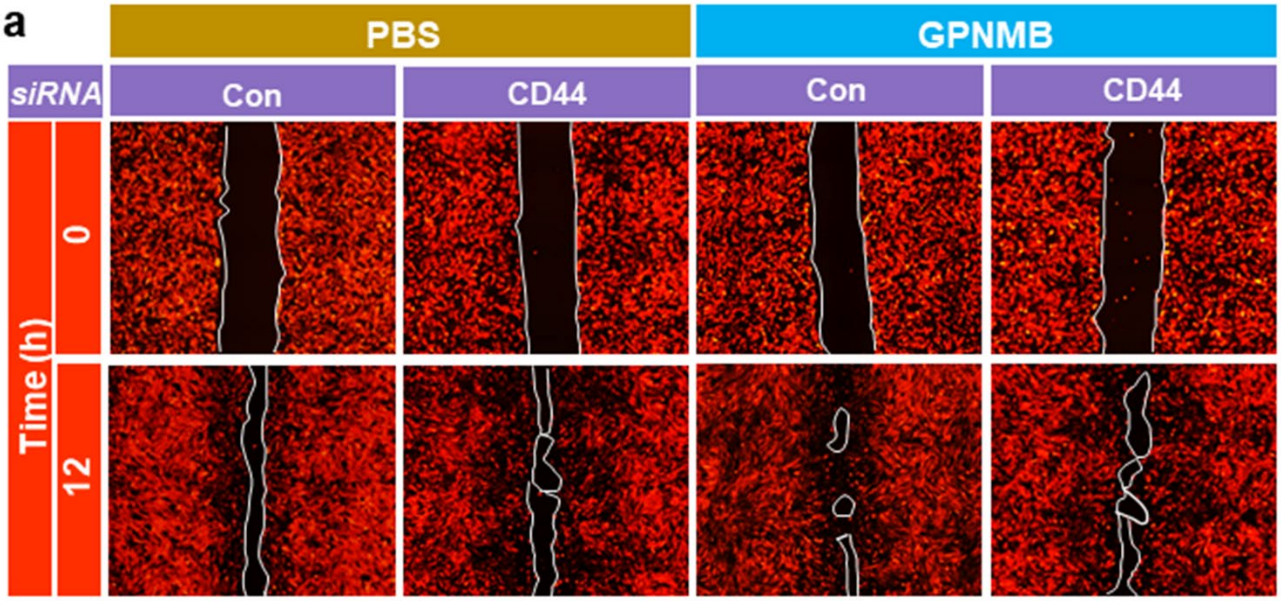

Supplementary Figure 8. The siRNA-induced CD44 knockdown in fibroblasts suppressed cell migration induced by GPNMB treatment. n≥5.

Supplementary Figure 9

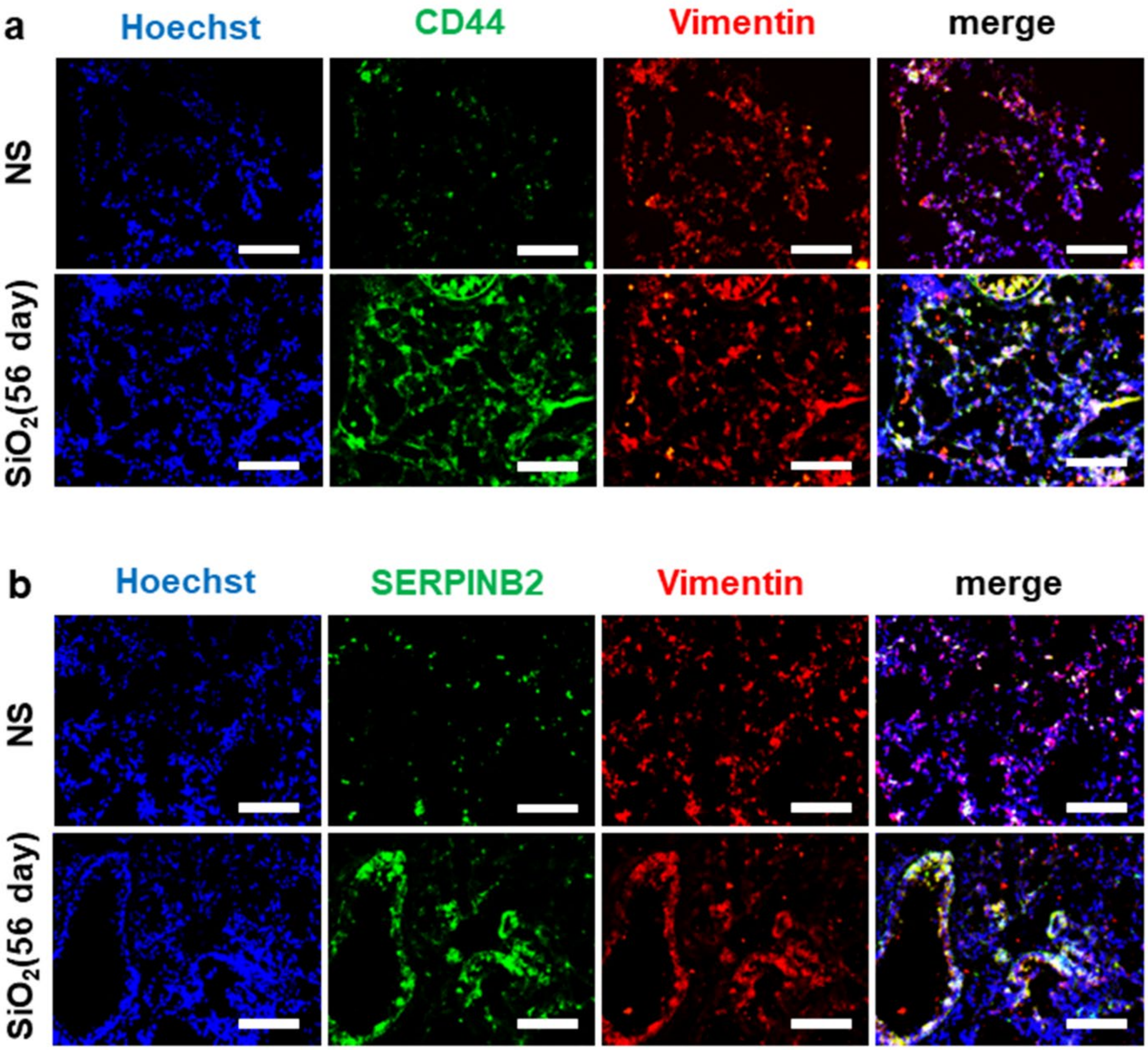

Supplementary Figure 9. The levels of CD44 and Serpinb2 in lungs of SiO2-treated mice. Compared with NS, CD44 (**a**) and Serpinb2 (**b**) increased after silica treatment, n≥5, scale bar=200 μm.

Supplementary Figure 10

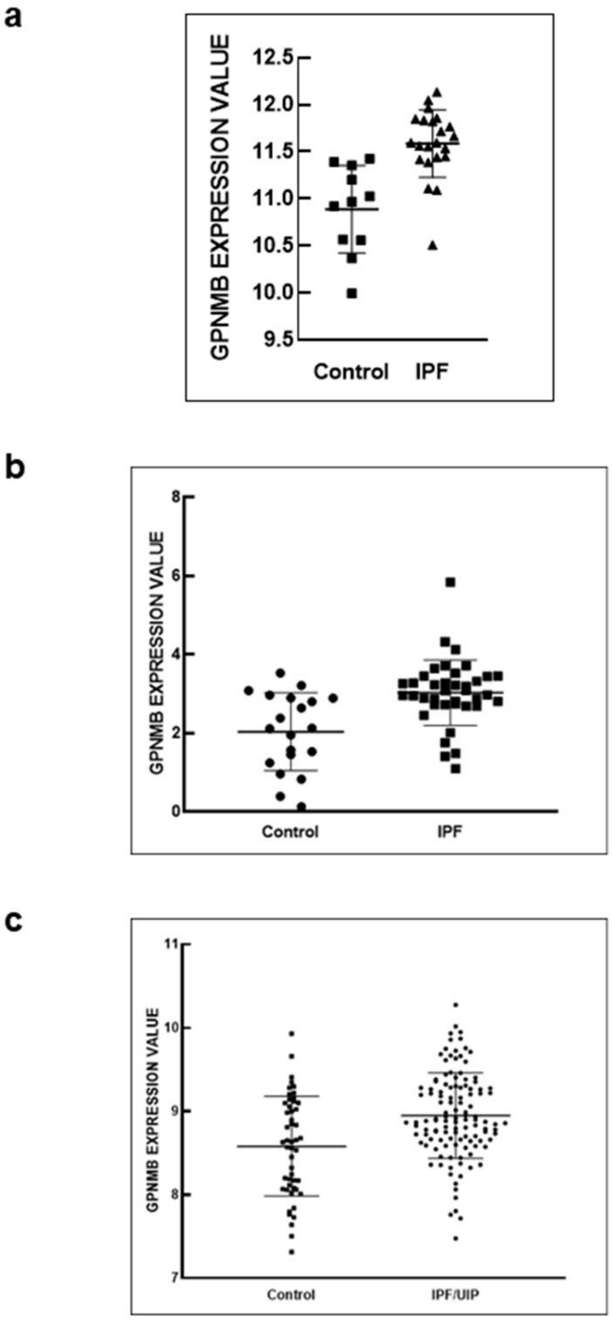

Supplementary Figure 10. Changes of GPNMB level in PF patients. (A-C) GPNMB levels were increased in PF patients.

Supplementary Figure 11

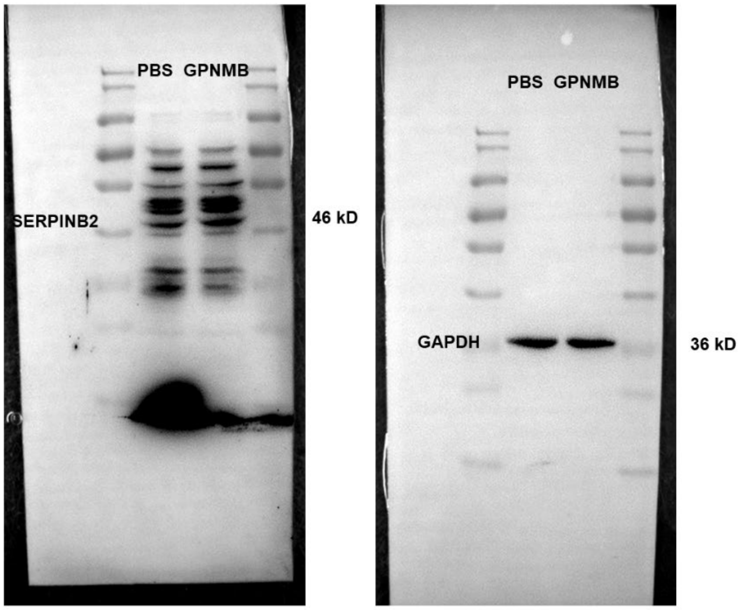

Supplementary Figure 11. The uncropped and unedited blots of Figure 7f.

Supplementary Figure 12

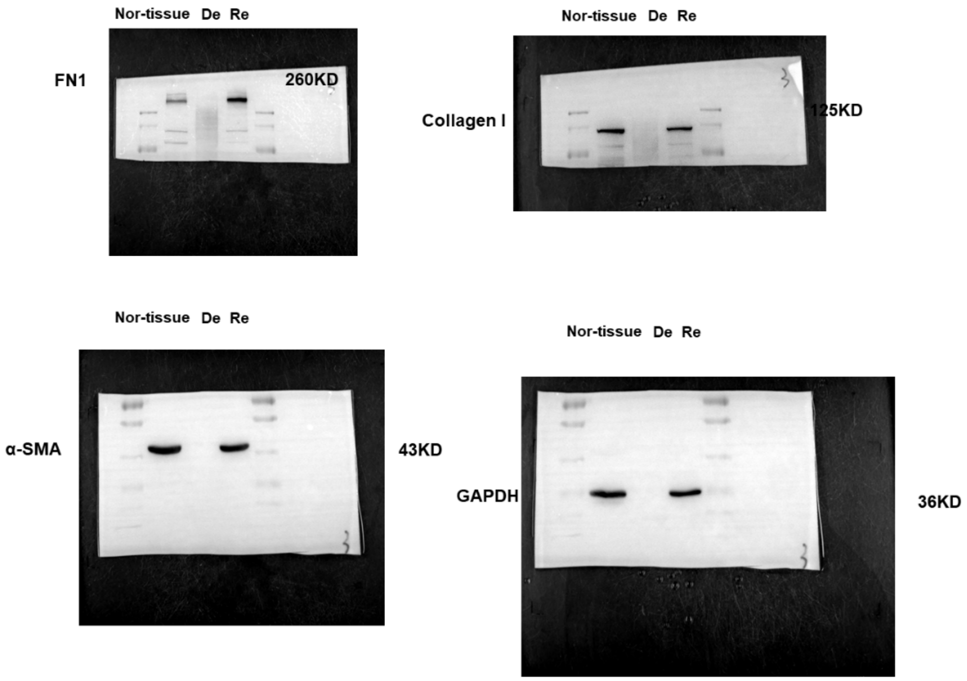

Supplementary Figure 12. The uncropped and unedited blots of Figure S1d.

Supplementary Figure 13

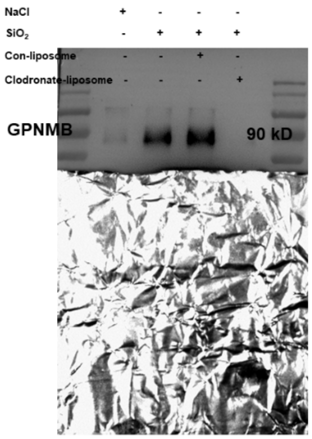

Supplementary Figure 13. The uncropped and unedited blots of Figure S5f.

Supplementary Figure 14

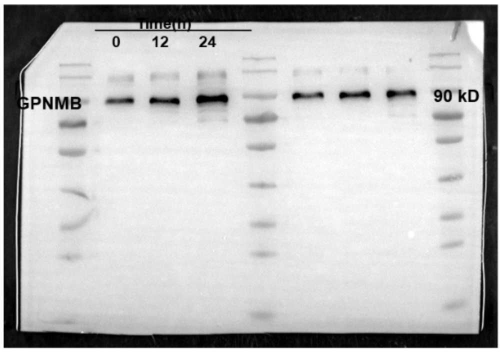

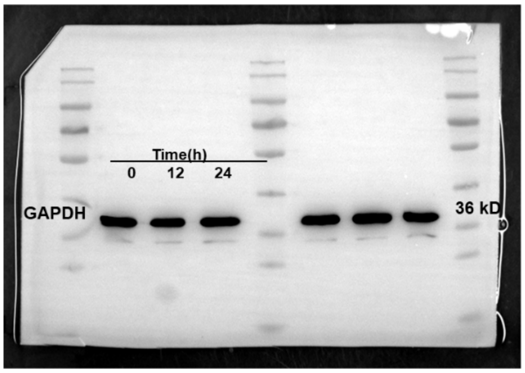

Supplementary Figure 14. The uncropped and unedited blots of Figure S6c.

Supplementary Figure 15

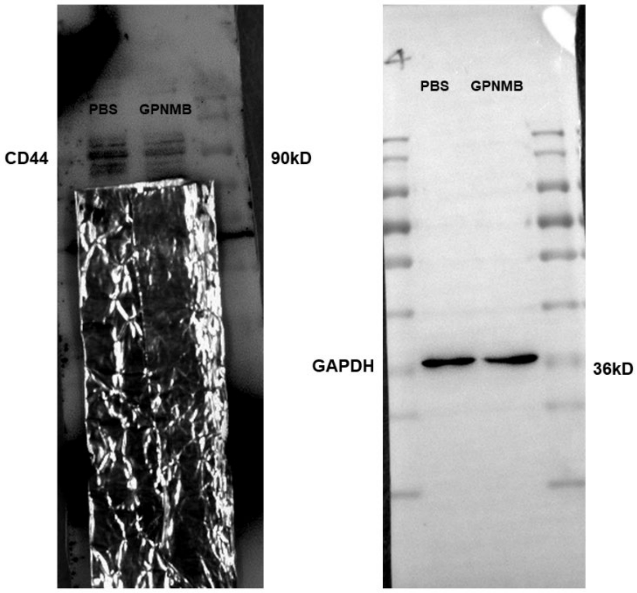

Supplementary Figure 15. The uncropped and unedited blots of Figure S6f.

Supplementary Table 1

The top 30 of upregulated proteins in the ECM of silicosis mice

Gene Name	Protein IDs	log2(FC)	FC
	P01878	3.321036	9.993819
Aprt	P08030	3.008259	8.045928
Sftpa1	P35242	2.968046	7.824758
Gpnmb	Q99P91	2.720419	6.590643
Sftpd	P50404	2.351895	5.104943
	P01636	2.290324	4.891659
	P06330;P01757;P01756	2.271521	4.82832
	P01631	2.190135	4.563481
Ctsc	P97821	2.141329	4.411681
	P18528;P18527;P18529;P18526;P18525	2.12091	4.349682
Sftpb	P50405	2.074996	4.213432
Cpxm1	Q9Z100	2.068522	4.194568
	P01635	2.063093	4.178812
Mzb1	Q9D8I1	2.058271	4.164868
C8a	Q8K182	1.924359	3.795681
Cpb2	Q9JHH6	1.901254	3.735378
Jchain	P01592	1.896641	3.723453
Cxcl15	Q9WVL7	1.876163	3.670974
Ccl6	P27784	1.849957	3.604894
Mcfd2	Q8K5B2	1.808746	3.503377
Igkc	P01837	1.790926	3.460368

Lilrb4	Q64281;Q61450	1.786369	3.449456
Mmp12	P34960	1.765188	3.399184
Npc2	Q9Z0J0	1.75585	3.377251
Ifit1	Q64282	1.745342	3.352744
Nmes1	Q810Q5	1.743898	3.349389
Napsa	O09043	1.728599	3.314059
Fabp5	Q05816	1.705139	3.260603
Hnrnpa1	P49312	1.701614	3.252648
Creld2	Q9CYA0	1.638904	3.114292

Supplementary Table 2

The upregulated mRNAs in fibroblasts between fibroblasts in different ECM

Gene ID	log2FC	FC
AW551984	3.731301	13.28109
Adora2a	3.467602	11.06247
Gm40881	2.774497	6.842374
Gm38529	2.343736	5.076153
Il21r	2.200539	4.596511
Gm2959	1.993214	3.981228
Bank1	1.969937	3.917509
Itgal	1.562543	2.95374
Gli1	1.56192	2.952466
Cd5	1.505363	2.83896
Serpinb2	1.390509	2.621711
Gm14434	1.310604	2.480454
Prkch	1.254639	2.386074
Satb1	1.15891	2.232887
Irf4	1.139386	2.202872
Ctla4	1.088483	2.126503

References

1. Zhang Z, Zhang F, An P, Guo X, Shen Y, Tao Y, et al. Ferroportin1 deficiency in mouse macrophages impairs iron homeostasis and inflammatory responses. *Blood* 2011; 118: 1912-1922.
2. Chen YC, Lai YS, Hsuuw YD, Chang KT. Withholding of M-CSF Supplement Reprograms Macrophages to M2-Like via Endogenous CSF-1 Activation. *Int J Mol Sci* 2021; 22(7): 3532
